# Tumor and peritumor radiomics analysis based on contrast-enhanced CT for predicting early and late recurrence of hepatocellular carcinoma after liver resection

**DOI:** 10.1186/s12885-022-09743-6

**Published:** 2022-06-17

**Authors:** Nu Li, Xiaoting Wan, Hong Zhang, Zitian Zhang, Yan Guo, Duo Hong

**Affiliations:** 1 Department of Breast Surgery, The First Hospital of China Medical University, No.155 Nanjing Road, Heping District, Shenyang, 110000 Liaoning China; 2 Department of Nuclear Medicine, Sun Yat-sen Memorial Hospital, Sun Yat-sen University, Guangzhou, 510000 China; 3 Department of Radiology, The First Hospital of China Medical University, No.155 Nanjing Road, Heping District, Shenyang, 110000 Liaoning China; 4 GE Healthcare, Beijing, China; 5 Department of Interventional Radiology, The First Hospital of China Medical University, No.155 Nanjing Road, Heping District, Shenyang, 110000 Liaoning China

**Keywords:** Radiomics, Hepatocellular carcinoma, Liver resection, Recurrence, Computed tomography (CT)

## Abstract

**Background:**

In China, liver resection has been proven to be one of the most important strategies for hepatocellular carcinoma patients, but the recurrence rate is high. This study sought to investigate the prognostic value of pretreatment tumor and peritumor contrast-enhanced CT radiomics features for early and late recurrence of BCLC stage 0-B hepatocellular carcinoma after liver resection.

**Methods:**

This study involved 329 hepatocellular carcinoma patients after liver resection. A radiomics model was built by using Lasso-Cox regression model. Association between radiomics model and recurrence-free survival was explored by using Harrell’s concordance index (C-Index) and receiver operating characteristic (ROC) curves. Then, we combined the radiomics model and clinical factors to establish a nomogram whose calibration and discriminatory ability were revealed.

**Results:**

Ten significant tumor and peritumor features were screened to build the radiomics model whose C-indices were 0.743 [95% CI, 0.707 to 0.778] and 0.69 [95% CI, 0.629 to 0.751] in the training and validation cohorts. Moreover, the discriminative accuracy of the radiomics model improved with peritumor features entry. The C-indices of the combined model were 0.773 [95% CI, 0.739 to 0.806] and 0.727 [95% CI, 0.667 to 0.787] in the training and validation cohorts, outperforming the radiomics model.

**Conclusions:**

The tumor and peritumor contrast-enhanced CT radiomic signature is a quantitative imaging biomarker that could improve the prediction of early and late recurrence after liver resection for hepatocellular carcinoma patients when used in addition to clinical predictors.

**Supplementary Information:**

The online version contains supplementary material available at 10.1186/s12885-022-09743-6.

## Background

Hepatocellular carcinoma (HCC) is one of the most common and life-threatening neoplasms worldwide. It is particularly prevalent in Asia and Africa [1]. In China, liver resection (LR) with curative intent has been proven to be one of the most important strategies for HCC patients [2], but the long-term prognosis remains unsatisfactory given the high rate of cancer recurrence of up to 60–70% in patients within 5 years after surgery [3]. Therefore, identifying risk factors for recurrence is of vital importance to improve postoperative long-term survival [4]. The Barcelona Clinic Liver Cancer (BCLC) staging system has been adopted and approved for guidance of HCC management [5]. This staging system has been proposed for prognostic prediction paired with treatment allocation, but its prognostic performance is simply based on stages. Furthermore, BCLC recommend that only patients with very early (BCLC stage 0) and early-stage (BCLC stage A) HCC should undergo LR, but many hepatobiliary centers in the world manage LR to patients with intermediate stage (BCLC stage B) HCC and achieve long-term survival [6–8]. Hence, the prediction of prognosis based on the BCLC staging system is far from satisfactory. Recently, a few statistical models have been developed to predict HCC recurrence [9], such as the Korean model [10], which was constructed based on preoperative clinical and postoperative pathologic characteristics; however, none of these models took tumor morphology into consideration.

Radiomics analysis can extract a large number of imaging features quantitatively, which could offer a cost-effective and non-invasive approach for individual medicine [11]. Some studies have employed radiomics to predict recurrence of HCC after LR; however, most of these studies paid attention to early recurrence (≤ 2 years) rather than late recurrence (> 2 years) [12–15]. In addition, although a few previous studies added peritumor areas that might harbor highly invasive tumor cells, the context of underlying cirrhosis was not considered [16, 17]. This study aimed to investigate whether tumor and peritumor radiomic analysis of contrast-enhanced computed tomography (CECT) could improve the prediction of early and late recurrence after LR for HCC patients.

## Methods

### Study population

**Fig. 1:**
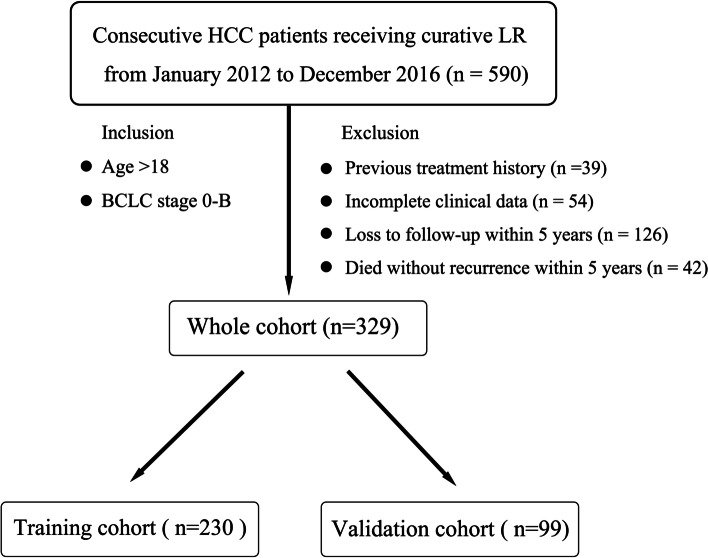
Flowchart of criteria for patient inclusion

**Fig. 2:**
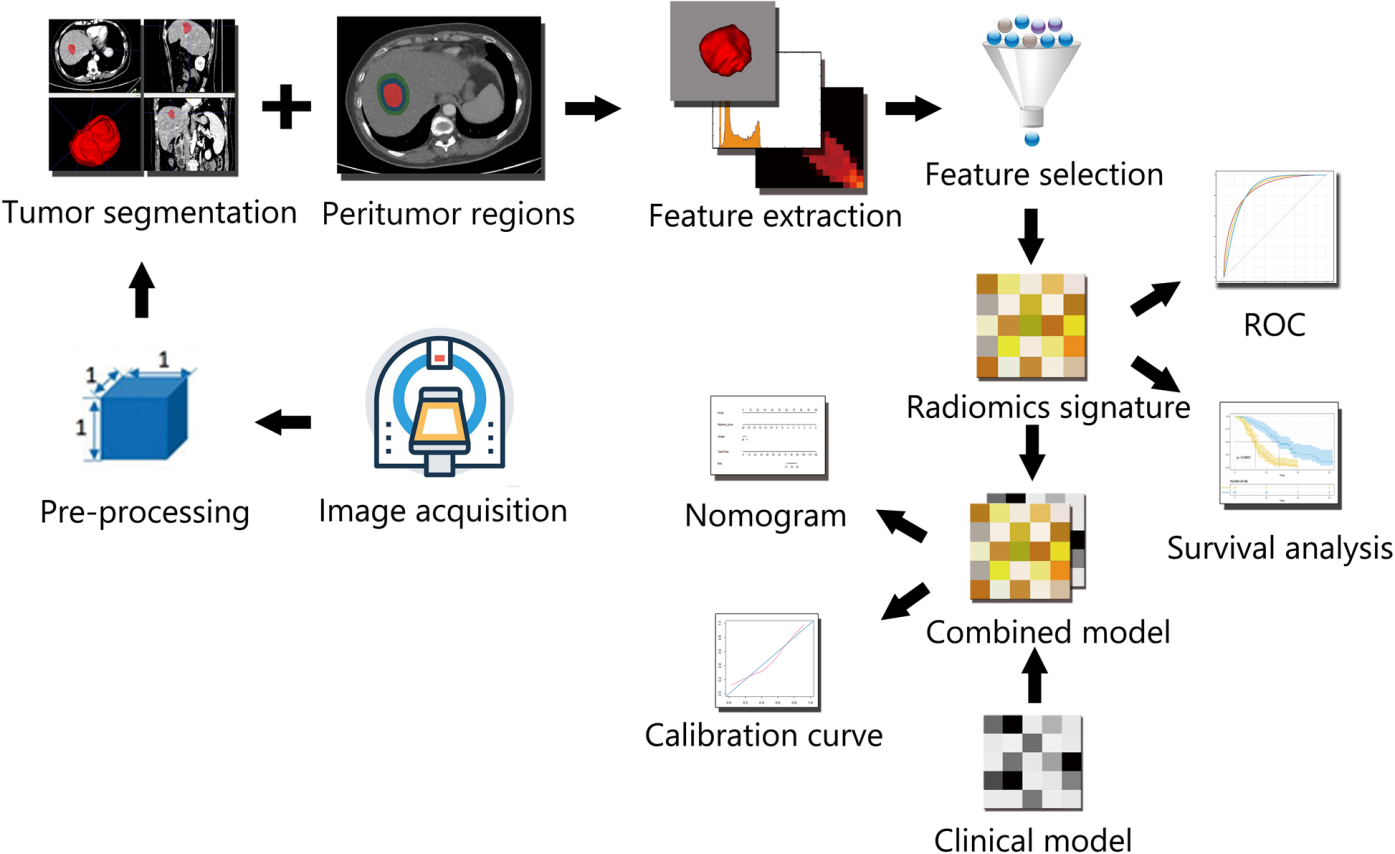
Workflow of the radiomics analysis

 Institutional review board approval was obtained for this retrospective study, and the requirement for the informed consent was waived. From January 2012 to December 2016, a total of 590 consecutive patients who were pathologically diagnosed with HCC and underwent CECT examination within 2 weeks before curative resection were recruited. The inclusion criteria were as follows: (1) age > 18 years; (2) BCLC stage 0-B. The exclusion criteria were as follows: (1) previous treatment history (ablation, TKI or transarterial chemoembolization) (*n* = 39); (2) incomplete clinical data (*n* = 54); and (3) loss to follow-up or death without recurrence within 5 years after LR (*n* = 168). Ultimately, 329 patients were included in this study (Fig. 1). The clinical factors included age, sex, hepatitis B virus (HBV), Child–Pugh liver function, BCLC classification, largest tumor diameter, tumor number, microvascular invasion (MVI) and laboratory values [albumin (ALB), total bilirubin (TBIL), alanine aminotransferase (ALT), prothrombin time (PT), a-fetoprotein (AFP)]. The patients were randomly divided into training cohort (*n* = 230) and validation cohort (*n* = 99) at a ratio of 7:3. Figure 2 illustrated the workflow of our study.

### Follow-up

Patients were followed up after LR, and HCC recurrence was screened by means of AFP and imaging examinations every 3 months. Recurrence-free survival (RFS) was defined as the time from the date of surgery to the date of first recurrence, metastasis, or last follow-up.

### Image acquisition and preprocessing

CECT was performed using the following equipment: Toshiba Aquilion One, Phillips Brilliance iCT 256 (Philips Medical Systems) or Toshiba Aquilion 64 (Toshiba Medical Systems) scanners. The scanning parameters were as follows: tube voltage, 120 kVp; tube current, 200–250 mA; detector collimation, 64, 256 or 320 × 0.625 mm; field of view, 350 × 350 mm; matrix, 512 × 512; reconstructed slice thickness, 2 mm. After a routine unenhanced scan, we injected contrast material intravenously at a flow rate of 3.0 mL/s to obtain CECT images. Hepatic arterial phase CT images were obtained 30 s after injection. Through preprocessing, images were resampled to a volume of 1.0 mm*1.0 mm *1.0 mm to standardize the voxel spacing.

### Tumor segmentation and feature extraction

Three-dimensional (3D) contours of the tumor regions of interest (ROIs) were delineated manually as gross-tumor regions (GTRs). All of the CT images were independently evaluated by two senior abdomen radiologists (9 and 15 years of experience) who were blinded to the clinical and pathological information. The radiologists delineated the boundaries of GTR on a transversal plane using ITK-SNAP (opensource software; version 3.4.0; www.itk-snap.org) software. The peritumor region (PTR) was defined as the parenchyma that fell within a 2-cm distance to the tumor boundary [18]. We outlined the tumor contours twice (each time for 1 cm) in three dimensions automatically using in-house software (Artificial Intelligence Kit, A.K., GE Healthcare) and modified the boundaries manually. Finally, two peritumor regions (PTR1 and PTR2) were acquired for each GTR. PTR1 (0–1 cm) represented a micrometastatic region; PTR2 (1–2 cm) represented the context of underlying cirrhosis. In patients with multiple (two or more) lesions, the largest lesion was segmented and used for subsequent analysis.

A total of 1188 radiomics features were generated automatically using A.K. from GTR and two PTRs (396 for each region). All radiomics features for each region could be divided into three groups: (a) morphological features (*n* = 9): describe the morphology of the ROI; (b) first-order features (intensity features, *n* = 42) : related to the distribution of the intensities of voxels within the ROI, ignoring the spatial interactions between them, and can be calculated from histogram analysis; and (c) texture features (*n* = 345) [19]: are able to quantify how pixels are positioned in relation to each other, e.g. grey level co-occurring matrix (GLCM) are co-occurring pixels in each defined direction and are counted and recorded into a matrix [20]. The details are provided in Fig. S1. Inter-/intraclass correlation coefficients (ICCs) were used to evaluate radiomics feature reliability [21]. To assess interobserver reproducibility, the ROIs of 30 randomly chosen images were performed by the two abdomen radiologists independently. To evaluate intra-observer reproducibility, the radiologists repeated the same procedure at one-month intervals. Features with ICC > 0.75 was considered to indicate good agreement and entered in next analysis.

### Feature selection and radiomics model building

Least absolute shrinkage and selection operator Cox regression analysis (LASSO-Cox) was utilized to select the most significant radiomic features. LASSO has the key advantage of simultaneously selecting important variables and estimating their effects on the outcome from large number of candidate predictors. This selection method was proposed for linear or generalized linear models initially, where the outcome was fully observed. Then it has been extended to the Cox model (LASSO-Cox) for censored time-to-event responses. Based on the optimal lambda value that was selected through a 10-fold cross-validations, a panel of prognostic radiomic features was determined and built a GTR-PTR1-PTR2 model in the training cohort. The Rad-score in radiomics model was calculated via a linear combination of the selected features that had been weighted by their respective coefficients, represented quantitative ROI characteristics of each patient. Then, the GTR model and GTR-PTR1 model were established in the same manner, and their discriminative power was compared with the GTR-PTR1-PTR2 model.

### Validation of radiomics model

1. High and low risk groups were classified according to the optimal Rad-score cut-point which was obtained by using X-tile software (version 3.6.1; Yale University School of Medicine, New Haven, Conn). Recurrence probabilities were estimated using the Kaplan-Meier method and compared by the log-rank test between the two subgroups.

(2) The receiver operating characteristic (ROC) curves and areas under the curves (AUCs) were employed to investigate the performance at different RFS time points of 1-, 2- and 5-year.

### Combined model construction and validation

Age, sex, hepatitis B virus (HBV), Child–Pugh liver function, BCLC classification, largest tumor diameter, tumor number, microvascular invasion (MVI) and laboratory values were analyzed by univariate Cox regression. Significant factors (*p* < 0.05) were chosen as candidates.

The combined model was constructed from the Rad-score and the candidate clinical factors by multivariate Cox regression; the significant factors with *p* < 0.05 were included in the model. A nomogram for 1-, 2- and 5-year RFS rate predictions was plotted according to the combined model. Then, the C-index and calibration curve were generated from the combined model.

### Statistical analysis

Descriptive statistics are presented as the median (interquartile range [IQR]) for continuous variables and the frequency (%) for categorical variables. To determine significant differences between the training and validation cohorts, continuity correction and Pearson chi-square tests were used. All statistical tests were two tailed, and a *p* value < 0.05 indicated a significant difference. All statistical analyses were performed using R statistical software version 3.6.2.

## Results

### Clinical characteristics

The clinical characteristics of the patients in the training and validation cohorts are listed in Table 1. The median age of the total cohort was 57 years (range from 23 to 83). The study included 262 (79.6%) male patients and 67 (20.4%) female patients. The median RFS was 33 months (range of 3–95 months). No differences in clinical characteristics were noted between the training dataset and the test dataset (all *p*-value < 0.05).

**Table 1:** Baseline characteristics for both cohorts

	Training cohort (*N* = 115)	Validation cohort (*N* = 50)	*p*
Sex/No. (%)			0.583^a^
Male	185 (80.4)	77 (77.8)	
Female	45 (19.6)	22 (22.2)	
Age	57 (49–64)	58 (51–63)	0.284^b^
Hepatitis B virus infection/No. (%)			0.618^a^
Positive	190 (82.6)	84 (84.8)	
Negative	40 (17.4)	15 (15.2)	
BCLC/No. (%)			0.329^a^
Early stage (0-A)	202 (87.8)	83 (83.8)	
Intermediate (B)	28 (12.2)	16 (16.2)	
Child’s class/No. (%)			0.326^a^
A	177 (77)	81 (81.8)	
B	53 (23)	18 (18.2)	
Largest tumor diameter (cm)	5.4 (3.5–7.7)	5.1 (3.1–7.6)	0.430^b^
Tumor number/(%)			0.079^a^
Single	197 (85.6)	77 (77.8)	
Multiple	33 (14.3)	22 (22.2)	
MVI/No. (%)			0.462^a^
Positive	202 (87.8)	84 (84.8)	
Negative	28 (12.2)	15 (15.2)	
AFP level (ng/mL)			0.673^a^
≤ 400	136 (59.1)	61 (61.6)	
> 400	94 (40.9)	38 (38.3)	
PT (S)	12.8 (12.3–13.5)	12.7 (12.1–13.3)	0.163^b^
TBIL (µmol/L)	13.9 (11.0-17.5)	12.8 (10.9–17.3)	0.156^b^
Albumin (g/L)	38.7 (35.8–40.8)	38.4 (36.0-40.4)	0.716^b^
ALT(U/L)	44 (28–64)	43 (17–67)	0.421^b^
Rad-score	-8.11 (-8.57–7.71)	-8.25 (-8.66–7.77)	0.217^b^

*BCLC* The Barcelona Clinic Liver Cancer staging system, *MVI *Microvascular invasion, *AFP *Alpha-fetoprotein, *ALB *Albumin, *ALT *Alanine aminotransferase, *PT *Prothrombin time, *TBIL *Total bilirubin

* *p* < 0.05

^a^ Pearson χ2 test

^b^ Mann–Whitney U test

### Feature selection and radiomics model building

The ICCs of 892 features with > 0.75 inter- and intraclass correlation results were reserved for further calculation. In the training cohort, 10 features were evaluated to build a radiomics model through the LASSO-Cox algorithm (Fig. S2): [1] MeanDeviation; [2] GLCMEnergy_angle135_offset1; [3] Percentile10 peri1; [4] GLCMEnergy_angle45_offset7 peri1; [5] GLCMEntropy_angle135_offset7 peri1; [6] ShortRunEmphasis_AllDirection_offset1_SD peri1; [7] ShortRunEmphasis_angle135_offset1 peri1; [8] ShortRunHighGrayLevelEmphasis_AllDirection_offset4_SD peri1; [9] LongRunLowGrayLevelEmphasis_angle0_offset1 peri2; [10] RunLengthNonuniformity_AllDirection_offset1_SD peri2. The formula of Rad-score is illustrated in Table S1.

## Validation of the radiomics model

For the training and validation cohorts, respectively, the C-indices of the radiomics model (GTR-PTR1-PTR2 model) were 0.743 [95% CI, 0.707 to 0.778] and 0.69 [95% CI, 0.629 to 0.751]; the C-indices of the GTR model were 0.694 [95% CI, 0.657 to 0.732] and 0.661 [95% CI, 0.598 to 0.725], the C-indices of the GTR-PTR1 model were 0.737 [95% CI, 0.701 to 0.772] and 0.673 [95% CI, 0.613 to 0.735].

**Fig. 3:**
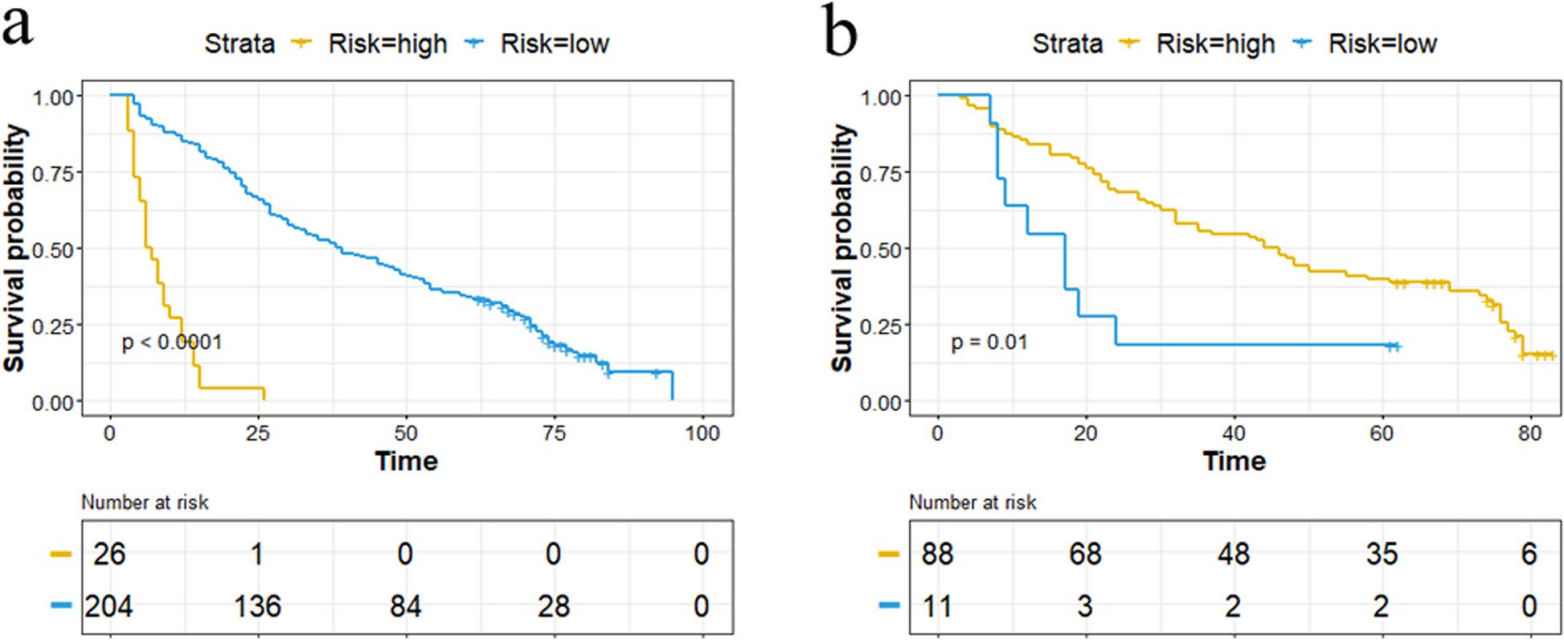
Kaplan–Meier curves for high and low risk groups. The radiomics model could help to stratify high and low risk patients effectively. **a** Training cohort. **b** Validation cohort

**Fig. 4:**
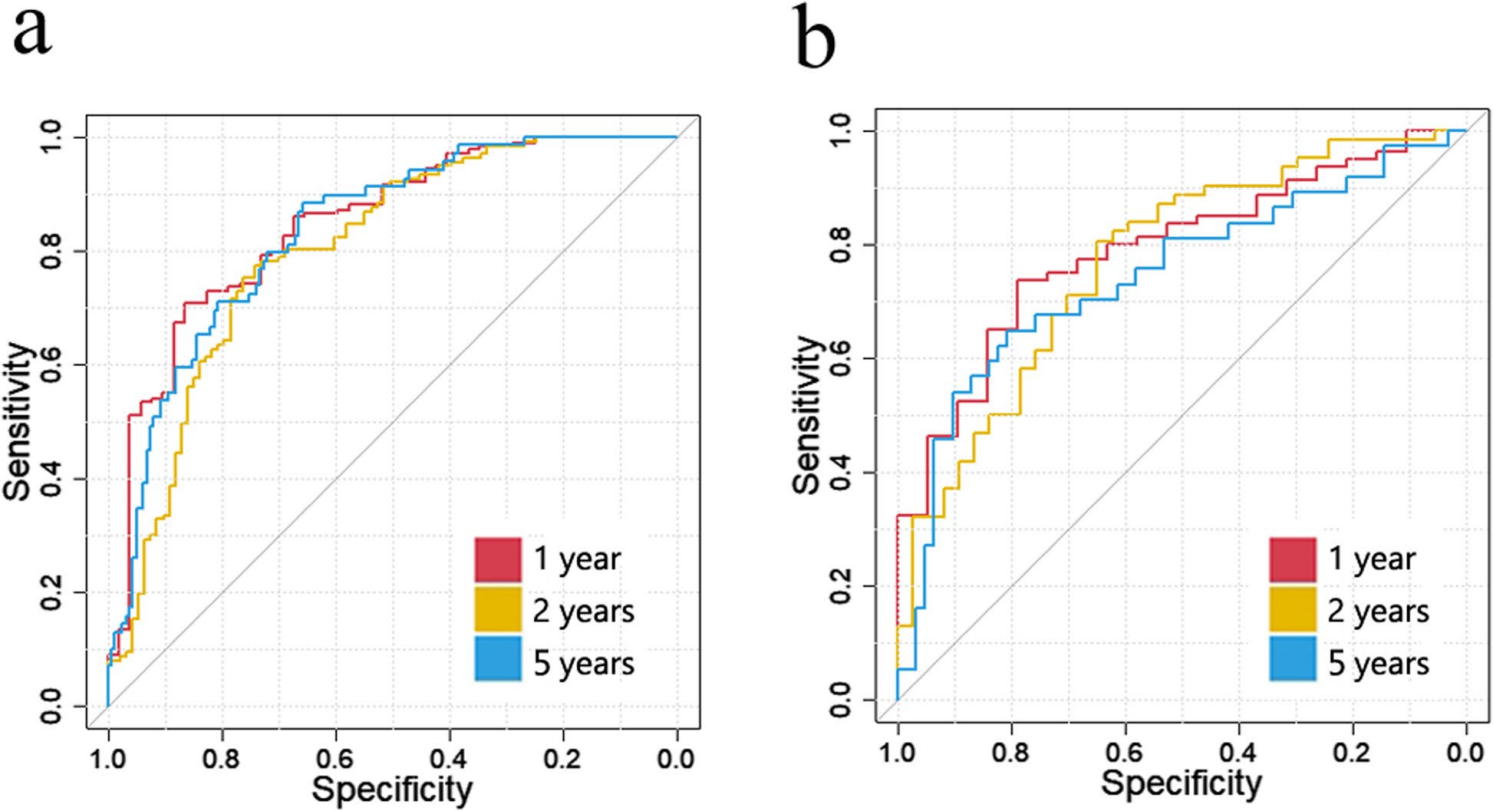
The ROC curves for 1-, 2- and 5-year RFS using the radiomics model. **a** Training cohort. **b** Validation cohort

1. High and low risk groups were classified according to the optimal Rad-score cut-point of -7.34 according to X-Tile. Kaplan–Meier analysis showed that patients belonging to the high-risk group had higher recurrence probabilities in the two cohorts (both *p* < 0.05), as shown in Fig. 3.

(2) The ROC curves of the two cohorts for 1-, 2- and 5-year RFS are plotted in Fig. 4. The AUCs for 1-, 2- and 5-year RFS were 0.845 [95% CI, 0.783–0.906], 0.801 [95% CI, 0.741–0.862], and 0.834 [95% CI, 0.779–0.888], respectively, in the training cohort, and 0.791 [95% CI, 0.689–0.892], 0.771 [95% CI, 0.675–0.867] and 0.745 [95% CI, 0.638–0.852], respectively, in the validation cohort.

## Combined model construction and validation

**Fig. 5:**
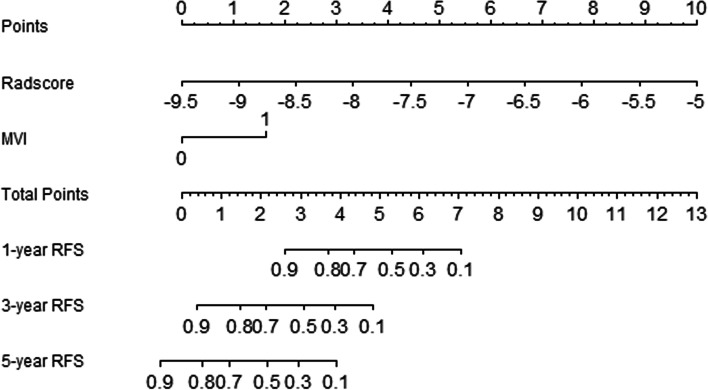
The constructed nomogram for combined model

**Fig. 6:**
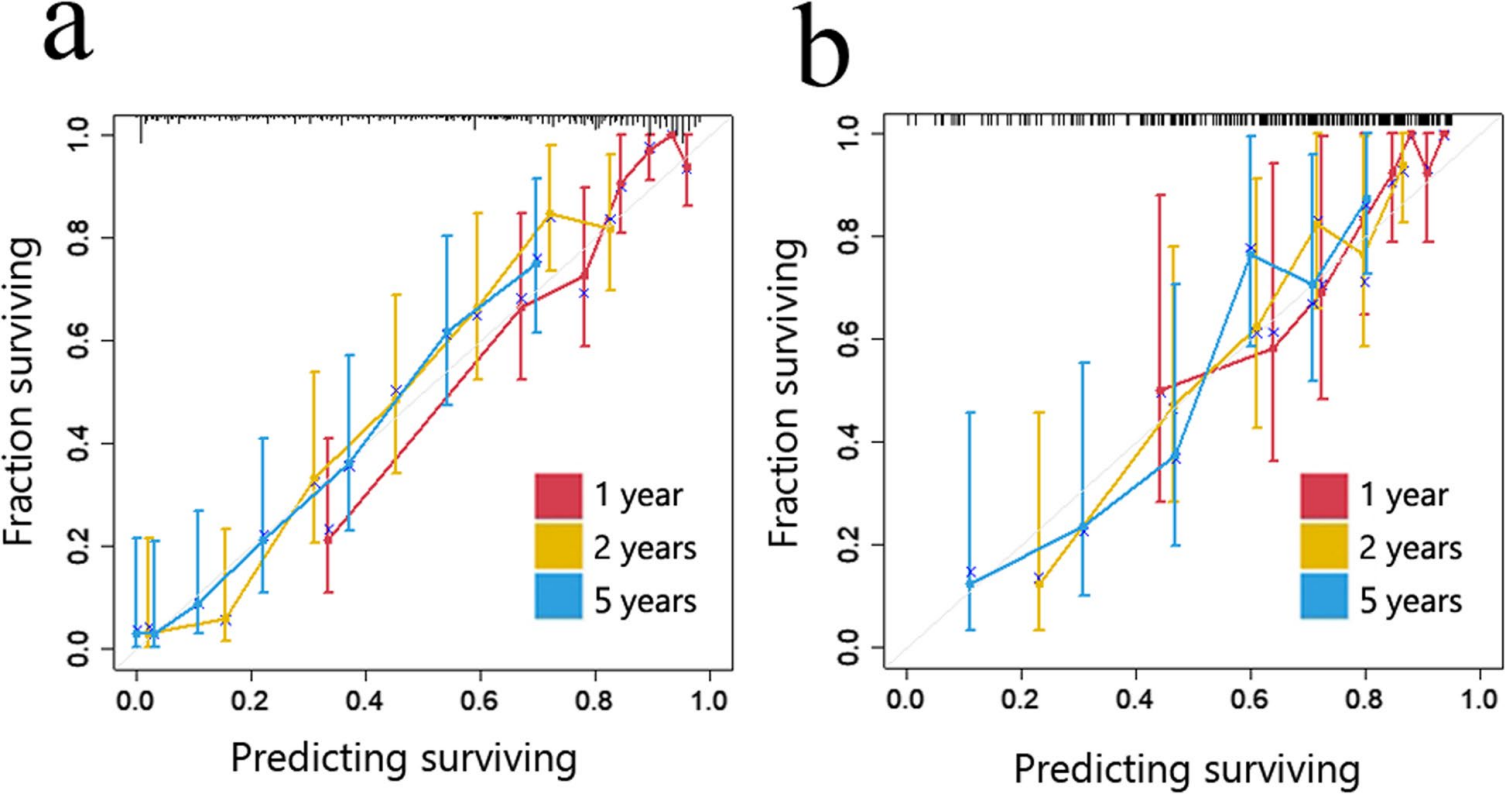
Calibration curves for nomogram. **a** Training cohort. **b** Validation cohort

The largest tumor diameter and MVI were significant factors (*p* < 0.05) in univariate Cox regression and tested with the Rad-score in multivariate Cox regression (Table 2). MVI and Rad-score were significant factors (*p* < 0.05) in the combined model, then a nomogram was constructed in the training cohort based on the combined model for the prediction of the 1-, 2- and 5-year RFS rates (Fig. 5). The C-indices of the combined model were 0.773 [95% CI, 0.739 to 0.806] in the training cohort and 0.727 [95% CI, 0.667 to 0.787] in the validation cohort. These values were higher than those of the radiomics model. Good calibrations for recurrence probabilities in the nomogram were observed in both cohorts (Fig. 6).

**Table 2:** Univariate and multivariate analyses for clinical factors and Rad-score

Risk factor	Univariate			Multivariate		
	HR	95% CI	*p*	HR	95% CI	*p*
Sex (M/F)	0.962	0.726 ~ 1.488	0.832			
Age (y)	0.998	0.985 ~ 1.013	0.874			
Hepatitis B (No /Yes)	1.25	0.851 ~ 1.836	0.256			
BCLC (0-A/B)	1.152	0.868 ~ 1.731	0.495			
Child’s class (A/B)	1.112	0.8 ~ 1.545	0.528			
Largest tumor diameter (cm)	1.125	1.076 ~ 1.175	< 0.01*	1.001	0.952 ~ 1.052	0.96
Tumor number (Single/ Multiple)	1.012	0.686 ~ 1.494	0.953			
MVI (Negative / Positive)	3.269	2.142 ~ 4.989	< 0.01*	3.092	2.043 ~ 4.68	< 0.01*
AFP level (ng/mL) (≤ 400/>400)	1.239	0.931 ~ 1.648	0.141			
PT (S)	0.967	0.853 ~ 1.095	0.595			
TBIL (µmol/L)	0.982	0.961 ~ 1.005	0.122			
Albumin (g/L)	0.985	0.949 ~ 1.022	0.418			
ALT(U/L)	0.999	0.994 ~ 1.006	0.943			
Rad-score	4.584	3.589 ~ 5.855	< 0.01*	4.669	3.554 ~ 6.136	< 0.01*

*BCLC* The Barcelona Clinic Liver Cancer staging system, *MVI *Microvascular invasion, *AFP *Alpha-fetoprotein, *ALB*: Albumin, *ALT *Alanine aminotransferase, *PT *Prothrombin time, *TBIL *Total bilirubin

*p < 0.05

## Discussion

In this retrospective study, a radiomics signature derived from 10 tumor and peritumor radiomic features of CECT was built for predicting postoperative early and late recurrence of BCLC stage 0-B HCC. In the validation cohort, the AUCs for 2- and 5-year RFS were 0.771 [95% CI, 0.675–0.867] and 0.745 [95% CI, 0.638–0.852], respectively. Then, a combined nomogram incorporating clinical characteristics and the radiomics signature was generated. The nomogram demonstrated good discrimination (C-index of 0.727 [95% CI, 0.667 to 0.787] in the validation cohort) and calibration. The combined model offered clinical utility, which might help clinicians design more effective treatment strategies tailored to the specific characteristics of individual patients and their disease [12].

At present, the BCLC system is regarded as the optimal staging system to guide treatment for HCC; however, the main limitation is that it recommends liver resection in very early (BCLC 0) and early stages (BCLC A). Some previous studies conducted research according to the BCLC system. For example, Gu et al. took early-stage HCC patients as the main object to investigate the association of HCC recurrence and radiomics features [22]. Thus, many patients, particularly in the intermediate stage, are excluded from the benefit of liver resection. The Chinese liver cancer staging (CNLC) system was established in 2017 and has been adopted ever since, which suggests resection in Ia, Ib (BCLC 0 + A) and IIa (BCLC B with 2–3 nodules, > 3 cm) patients, even in selects patients with IIb (BCLC B with ≥ 4 nodules) and IIIa (BCLC C with vascular invasion) HCC [23]. Our center manages LR according to CNLC. In this study, we included 44 (13.4%) BCLC B patients in accordance with real world conditions in China. However, BCLC C patients were excluded because ending vasculature full of tumor thrombus was difficult to delineate precisely.

In our study, the peritumor region was divided according to resection margin, which previous studies ignored [16, 17]. Diamantis et al. examined the effect of resection margin on the incidence of recurrence among patients undergoing hepatectomy and found that wide resection margins (> 1 cm) were associated with better RFS than narrow margins (< 1 cm), as micrometastases might be present in the 1 cm tumor periphery. Therefore, PTR1 (0–1 cm) represented a micrometastatic region; PTR2 (1–2 cm) represented the context of underlying cirrhosis, which was also related to HCC recurrence, especially late recurrence [24]. We constructed all 3 radiomics models for GTR, GTR + PTR1 and GTR + PTR1 + PTR2 and observed that the C-indices increased when PTRs were entered in a step-by-step manner. The C-indices of the GTV, GTV + PTR1 and GTV + PTR1 + PTR2 models were 0.661 [95% CI, 0.598 to 0.725], 0.673 [95% CI, 0.613 to 0.735] and 0.69 [95% CI, 0.629 to 0.751], respectively, in the validation cohort, indicating that PTRs could improve the discriminative accuracy of the radiomics model.

This study has some limitations. First, we presented a retrospective study design within a single institute, so selection bias may inevitably exist. In addition, the scanners and standard of hepatectomy might lack generality. Second, radiomic features were derived from manual segmentation by radiologists, which can be influenced by observers’ subjective trends. Third, most patients with HCC in China have a background of HBV-related cirrhosis. However, in the US and Europe, hepatitis C and excessive alcohol use are the main etiological factors of HCC [25]. Our study requires an external validation cohort to make the results more convincing, and the inclusion of such information is planned in our future studies.

## Conclusions

In summary, the tumor and peritumor CECT radiomic signature is a quantitative imaging biomarker that could improve the prediction of early and late recurrence after LR for HCC patients when used in addition to clinical predictors.

## Supplementary Information

**Additional file 1.**

**Additional file 2.**

**Additional file 3.**

## Data Availability

All data generated or analysed during this study are included in supplementary material of this published article.

## Abbreviations

AFP: Alpha-fetoprotein
ALB: Albumin
ALT: Alanine aminotransferase
AUC: Areas under the curve
BCLC: The Barcelona Clinic Liver Cancer staging system
C-INDEX: Harrell’s concordance index
CECT: Contrast-enhanced computed tomography
GTR: Gross-tumor region
HCC: Hepatocellular carcinoma
HBV: Hepatitis B virus
LR: Liver resection
MVI: Microvascular invasion
PT: Prothrombin time
PTR: The peritumor region
PACS: Picture Archiving and Communication System
RFS: Recurrence-free survival
ROC: Receiver operating characteristic
TBIL: Total bilirubin
