# Supplementary material for: Tumor and peritumor radiomics analysis based on contrast-enhanced CT for predicting early and late recurrence of hepatocellular carcinoma after liver resection
Source: BMC Cancer. 2022 Jun 17;22:664. doi: 10.1186/s12885-022-09743-6 (PMC9205126; doi:10.1186/s12885-022-09743-6)
Supplement: Supplementary file 1 — Additional file 1. [file 12885_2022_9743_MOESM1_ESM.docx]

Table S1 Selected features and their coefficients

| NO. | Coefficients | Features |
| --- | --- | --- |
| 1 | 1.199145e+03 | MeanDeviation |
| 2 | 3.594441 | GLCMEnergy_angle135_offset1 |
| 3 | -7.537341e-03 | Percentile10 peri1 |
| 4 | 2.575539e+01 | GLCMEnergy_angle45_offset7 peri1 |
| 5 | -4.980320e-01 | GLCMEntropy_angle135_offset7 peri1 |
| 6 | 1.597034e+02 | ShortRunEmphasis_AllDirection_offset1_SD peri1 |
| 7 | -3.513647 | ShortRunEmphasis_angle135_offset1 peri1 |
| 8 | 1.406950e-06 | ShortRunHighGrayLevelEmphasis_AllDirection_offset4_SD peri1 |
| 9 | -4.123415e-01 | LongRunLowGrayLevelEmphasis_angle0_offset1 peri2 |
| 10 | 1.260582e-10 | RunLengthNonuniformity_AllDirection_offset1_SD peri2 |
