# Supplementary material for: Tumor and peritumor radiomics analysis based on contrast-enhanced CT for predicting early and late recurrence of hepatocellular carcinoma after liver resection
Source: BMC Cancer. 2022 Jun 17;22:664. doi: 10.1186/s12885-022-09743-6 (PMC9205126; doi:10.1186/s12885-022-09743-6)
Supplement: Supplementary file 2 — Additional file 2. [file 12885_2022_9743_MOESM2_ESM.docx]

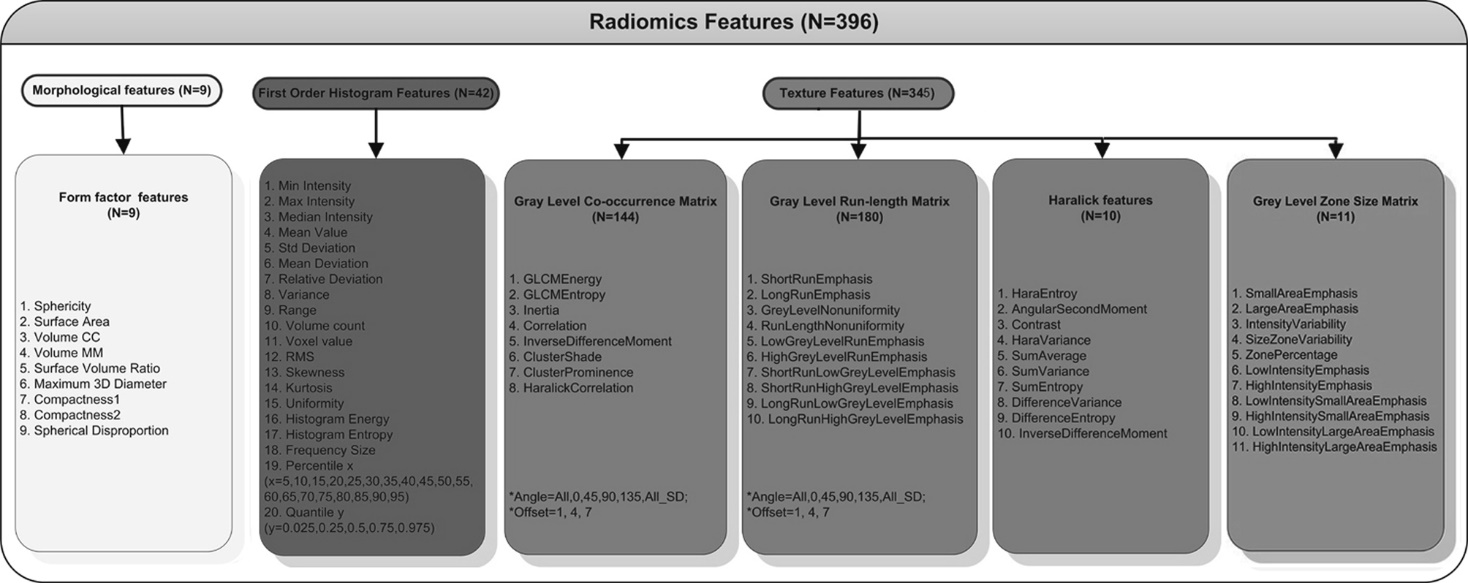


Fig. S1. Details of radiomics features extracted from CT images.


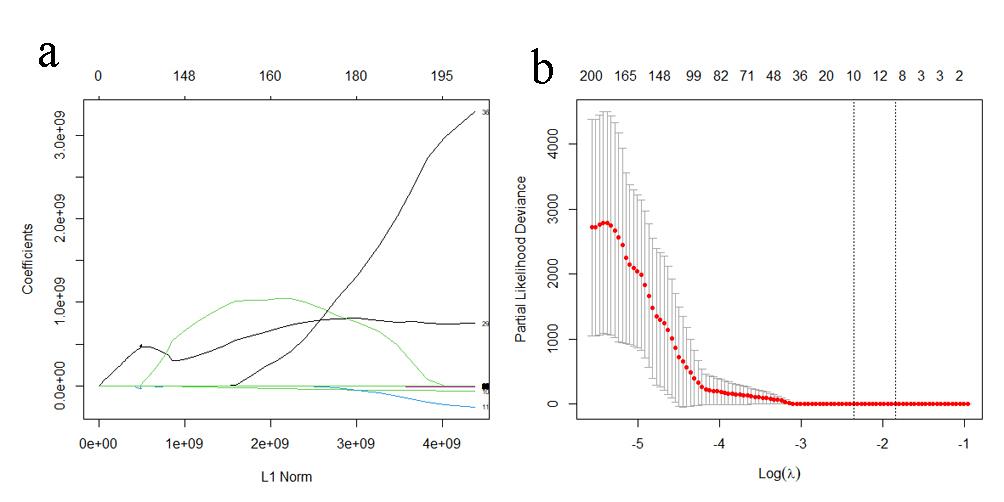


Fig. S2. LASSO-Cox regression for feature selection.
